# Cone-Beam Computed Tomographic Assessment of Mandibular Condylar Position in Patients with Temporomandibular Joint Dysfunction and in Healthy Subjects

**DOI:** 10.1155/2015/301796

**Published:** 2015-11-23

**Authors:** Maryam Paknahad, Shoaleh Shahidi, Shiva Iranpour, Sabah Mirhadi, Majid Paknahad

**Affiliations:** ^1^Oral Radiology Department, Dental School, Shiraz University of Medical Sciences, Shiraz 7145613466, Iran; ^2^Prevention of Oral and Dental Disease Research Center, Dental School, Shiraz University of Medical Sciences, Shiraz 7145613466, Iran; ^3^Radiology Department, Medical School, Shiraz University of Medical Sciences, Shiraz 7145613466, Iran

## Abstract

*Statement of the Problem*. The clinical significance of condyle-fossa relationships in the temporomandibular joint is a matter of controversy. Different studies have evaluated whether the position of the condyle is a predictor of the presence of temporomandibular disorder.* Purpose*. The purpose of the present study was to investigate the condylar position according to gender in patients with temporomandibular disorder (TMD) and healthy controls using cone-beam computed tomography.* Materials and Methods*. CBCT of sixty temporomandibular joints in thirty patients with TMD and sixty joints of thirty subjects without TMJ disorder was evaluated in this study. The condylar position was assessed on the CBCT images. The data were analyzed using Pearson chi-square test.* Results*. No statistically significant differences were found regarding the condylar position between symptomatic and asymptomatic groups. Posterior condylar position was more frequently observed in women and anterior condylar position was more prevalent in men in the symptomatic group. However, no significant differences in condylar position were found in asymptomatic subjects according to gender.* Conclusion*. This study showed no apparent association between condylar positioning and clinical findings in TMD patients.

## 1. Introduction 

The temporomandibular joint (TMJ) is one of the most complex joints in the body which is located between the mandibular condyle and the temporal bone [1, 2]. The radiographic joint space is a radiolucent area between the mandibular condyle and the temporal bone [3]. Joint space measurements were introduced by Ricketts to describe condylar position [4]. The condylar position can be determined by the relative dimensions of the radiographic joint spaces between the glenoid fossa and the mandibular condyle [3].

The clinical significance of condyle-fossa relationships in the temporomandibular joint is a matter of controversy [5]. Some studies have suggested an association between eccentric condylar position and temporomandibular disorder (TMD) [6–9]. These studies have suggested therapeutic procedures to optimize the condylar position in some patients [6, 10, 11]. However, other studies failed to demonstrate significant association between the condylar positioning and the incidence of TMD [12, 13].

Various radiographic methods have been used in previous studies to determine condylar position such as plain film radiography, conventional tomography, computed tomography, cone-beam tomography, and magnetic resonance imaging [5, 14–18]. Cone-beam computed tomography (CBCT) is the modality of choice for the assessment of temporomandibular osseous structures [19]. In the present study, the observers have used CBCT to study condylar positioning. The aim of the present study was to investigate the condylar position according to gender in patients with TMD and healthy controls using CBCT.

## 2. Materials and Methods

This study was carried out at the Department of Maxillofacial Radiology at Shiraz Dental University in Shiraz, Iran. An expert radiologist examined the participants and divided them into two groups including symptomatic group and asymptomatic group. The symptomatic group consisted of 30 patients (20 females and 10 males) aged 20 to 42 years (mean 33/4 years) with clinical signs and symptoms of TMD such as joint pain, muscle pain, mouth-opening limitation, joint noise (click or crepitation), and nonharmonic movements of the joint who were referred to the Department of Maxillofacial Radiology for the treatment of TMDs and required CBCT for more investigation. The asymptomatic group consisted of 30 adults (18 females and 12 males) who had no temporomandibular symptoms and no history of occlusal equilibration or masticatory disorders referred to our department for reasons other than TMJ problems. The age of the patients in the control group ranged from 15 to 34 years (mean 24 years). In the control group, the patients who had any evidence of TMD in clinical or radiological examination were excluded from the present study. In both groups, the exclusion criteria were the presence of any congenital abnormalities and/or any systemic disease which could affect joint morphology such as rheumatoid arthritis.

All the participants took part voluntarily in this study and the written consent forms were taken from each of them after being informed about the nature of the study in detail. The study was approved by the local Ethical Committee of Shiraz Dental School.

### 2.1. CBCT of the TMJ

The CBCT scans of bilateral TMJs were performed by a NewTom VGi (QR Srl, Italy) with a field of view 15 cm × 15 cm. The exposure factors were 120 kv, 5 mA, and exposure time of 5 seconds. The subjects were standing and biting their teeth into maximum intercuspal position. Their heads were positioned with the Frankfurt plane parallel to the floor.

### 2.2. Condylar Position Assessment

**Figure 1:**
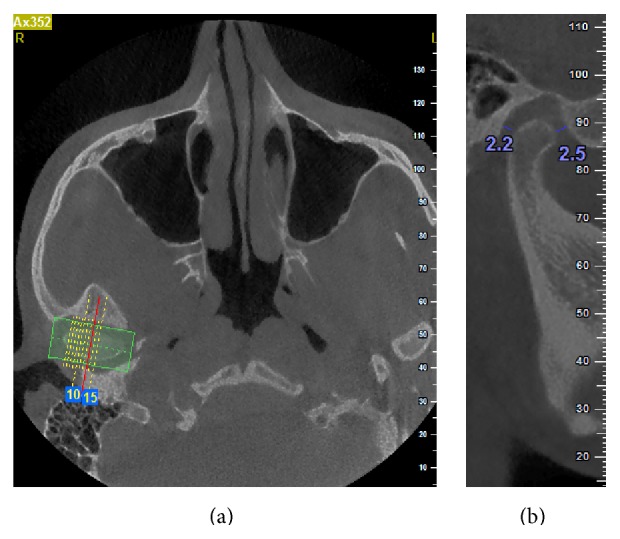
Linear measurement of anterior (*A*) and posterior (*P*) subjective closest joint spaces in a sample patient. (a) Axial view; (b) sagittal view.

The axial view, in which the condylar process had the widest mediolateral diameter, was chosen as the reference view for secondary reconstruction. On this selected axial view, a line parallel to the long axis of the condylar process was drawn and lateral slices were reconstructed with 0.5 mm slice interval and 0.5 mm thickness (Figure 1(a)). On the central sagittal section, an expert maxillofacial radiologist measured the values of the narrowest posterior (*P*) and anterior (*A*) joint space accurately using NewTom NNT analysis software (Figure 1(b)). Condylar position was expressed by the following formula according to the method of Pullinger and Hollender [20]:

(1) $$\begin{array}{c} \text{condylar ratio}=\frac{P-A}{P+A}\times 100. \end{array}$$

**Figure 2:**
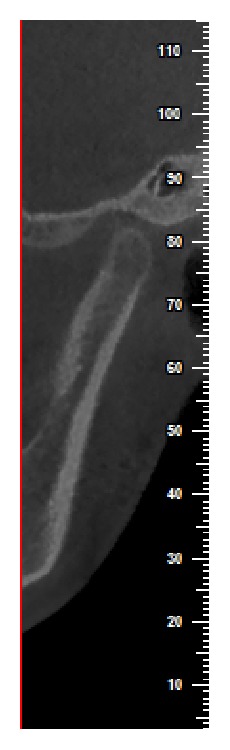
Posterior condylar position in a sample patient in the symptomatic group.

**Figure 3:**
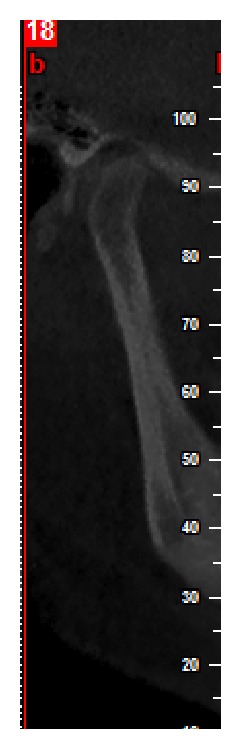
Anterior condylar position in a sample patient in the symptomatic group.

The position of the condyle was considered concentric if the ratio was within ±12%. If the ratio was smaller than −12%, the condylar position was considered posterior and if the ratio was greater than +12%, the condyle was considered in an anterior position (Figures 2 and 3).

### 2.3. Statistical Analysis

All data were analyzed with the SPSS program (SPSS 15.0, IBM, Chicago, IL, USA). The statistical analysis was performed using Pearson chi-square test to compare the condylar positions between two groups at the significance level of 0.05. To assess the significance of any errors during measurement, all images were revaluated over one-week interval. The mean difference between the first and second measurement was analyzed using paired *t*-test. The level for significance was set at *P* < 0.05.

## 3. Results

There were no significant differences between dual measurements. The means of these two measurement values were used to minimize the error in identifying the reference points.

In the asymptomatic group, the frequency of posterior position was 25%, concentric position 38.5%, and anterior position 36.7%. In the symptomatic objects the incidence of posterior condylar position was 38.3%, concentric position 36.7%, and anterior position 35% (Table 1). There was no significant difference between the symptomatic and the asymptomatic groups for condylar position (*P* value = 0.22). Distribution of the condylar position in the symptomatic and asymptomatic groups according to gender is summarized in Table 2. No significant differences in condylar position between men and women were found in the asymptomatic subjects (*P*  value = 0.757). The condylar position in the symptomatic group was significantly different in men and women (*P* value < 0.05) (Table 2). Posterior condylar position is significantly more prevalent in women (50%) and anterior condylar position more prevalent in men (55%).

## 4. Discussion

The clinical significance of condyle-fossa relationships in the temporomandibular joint is a matter of controversy [5]. Some studies have suggested eccentric condylar position is associated with temporomandibular disorder [6–9]. The aim of this study was to evaluate the condylar position according to gender in patients with TMD and healthy controls using CBCT.

Different radiographic techniques including conventional radiography [15], conventional tomography [18], computed tomography [1], MRI [14, 16, 17], and cone-beam computed tomography [5, 14, 21] have been used to study the condylar position in the glenoid fossa and the articular eminence morphology. Previously conventional radiography, especially transcranial radiography, has been used to assess condylar position and morphology [7]. However, transcranial radiographs only represent the lateral third of the condyle. Therefore the reliability of these radiographs or assessing condylar position is questioned. Some researchers used conventional tomography to evaluate condylar position in the glenoid fossa [20]. However because slice thickness is large ranging between 1.0 and 3.0 mm, it does not represent the margins of joint structure as clearly as CT and CBCT [22].

The recently developed CBCT represents the joint structures with high accuracy which produces submillimeter spatial resolution as high as or even superior to spiral CT [23, 24]. Kobayashi et al. reported that the measurement error in CBCT was significantly less than spiral CT [24]. The bony component can be visualized in 3 planes without any superimposition, distortion, or magnification [25, 26]. CBCT has the advantage of reduced radiation dose and shorter scanning time compared with CT [27]. Therefore, CBCT has been used in the present study.

In studies that used transcranial radiographs actually the most lateral part of the joint is evaluated. Rammelsberg et al. selected three tomograms including central, 3 mm more lateral, and 3 mm more medial and measured data in tomograms [28]. Ikeda and Kawamura evaluated joint spaces on the central cuts of joints within 3.5 mm range medially and laterally to the central cut in CBCT [29]. They found that landmark identification outside this range was default because of the glenoid fossa anatomy. They also suggested that there were not significant differences in the joint spaces in this section [29]. Therefore we only considered the central slice of sagittal section of condyles in order to simplify analyzing the data.

There is a controversy over the clinical significance of condylar position [5]. Many studies have reported nonconcentric condylar position in association with disk displacement [14, 17], osteoarthritic changes [5], remodeling of the articular eminence and the condyle [30], and predisposition to arthrosis [31]. Nonconcentric condylar positioning is seen in one-third to one-half of asymptomatic volunteers [3]. On the other hand, concentric positioning in patients with TMD has high prevalence [32]. Aggressive condylar repositioning therapies are frequently performed to reestablish the mandibular condyle in an optimal position [6, 10]. However, according to the present study, condylar eccentricity is not a sufficient evidence for diagnosis of TMD and besides the evaluation of TMJ clinical symptoms and assessment of condylar eccentricity, additional investigations are required before a therapeutic change is performed.

Some studies represented no significant association between condylar positioning and clinical findings [12, 33, 34]. However, many studies showed significant difference in the condylar positions in patients with TMD and asymptomatic subjects [27, 35]. Cho and Jung found concentric condylar position was more common in the asymptomatic group and posterior condylar position was more frequent in the symptomatic group [5]. Paknahad and Shahidi reported posteriorly seated condyles in patients with severe TMD and anteriorly and concentric seated condyles in patients with mild to moderate TMD [36]. Lelis et al. evaluated the condyle-mandibular fossa relationship in young individuals with intact dentitions and compared it to that between individuals with and without symptoms of temporomandibular disorder using CBCT [37]. They concluded that the presence or absence of temporomandibular disorder was not correlated with the condyle position in the temporomandibular joint which was similar to our findings.

In some previous studies asymptomatic groups represented more posterior condylar position in women and more anterior positions in men [20, 38]. Madsen found in the transcranial radiographs of asymptomatic adults that women and men were more likely to present posterior and anterior condylar positioning, respectively [39]. However in the present study, no significant difference in condylar position was found between men and women in asymptomatic subjects. Similarly some previous studies found no significant sex difference in condylar position joint spaces in normal joints [35, 40]. Ikeda and Kawamura found no significant sex difference in joint spaces, using CBCT in symptom-free subjects [29].

On the other hand in the symptomatic group posterior condylar position in women and anterior position in men were noticed. Some authors have reported an association between posterior condylar positioning and internal derangement [14, 16]. Higher incidence of posterior condylar position in women may be the etiological factor for preponderance of TMD and disk instability in women.

In this study the subjects who had history of occlusal therapy, prosthodontics treatment, and any systemic disorders such as rheumatoid arthritis were not included because these factors could affect the condylar morphology and position.

The present study did not demonstrate any significant differences in condylar position between symptomatic and asymptomatic groups. However several different factors such as radiographic technique used, accuracy of clinical examination, sample size, and the method of condylar position measurement can influence the results. Therefore, further investigations for assessing the correlation between temporomandibular disorder and condylar position are necessary.

## Figures and Tables

**Table 1:** Distribution of condyle position in the symptomatic and asymptomatic group.

Group	Condylar position			*P* value
	Posterior	Concentric	Anterior	
Asymptomatic	15 (25.0%)	23 (38.3%)	22 (36.7%)	0.22
Symptomatic	23 (38.3%)	16 (26.7%)	16 (26.7%)	

**Table 2:** The condylar position in the symptomatic and asymptomatic groups according to gender.

Group	Sex	Condylar position			*P* value
		Posterior	Concentric	Anterior	
Asymptomatic	Female	10 (27.8%)	14 (38.9%)	12 (33.3%)	0.757
	Male	5 (20.8%)	9 (37.5%)	10 (41.7%)	

Symptomatic	Female	20 (50%)	10 (25%)	10 (25%)	0.020^*∗*^
	Male	3 (15%)	6 (30%)	11 (55%)	

^*∗*^A *P* value less than 0.05 was considered statistically significant.
